# Environing as Embodied Experience—A Study of Outdoor Education as Part of Physical Education

**DOI:** 10.3389/fspor.2021.768295

**Published:** 2021-11-12

**Authors:** Suzanne Lundvall, Ninitha Maivorsdotter

**Affiliations:** ^1^Department of Movement, Culture and Society, Swedish School of Sport and Health Sciences, Stockholm, Sweden; ^2^University of Gothenburg, Gothenburg, Sweden; ^3^School of Health Sciences, University of Skövde, Skövde, Sweden

**Keywords:** physical education, environing, practical epistemology analysis, outdoor education, diversity, peri-urban nature, aesthetic experience

## Abstract

The development of a re-understanding or re-investigation of body pedagogy is currently prominent in the field of physical education (PE) and sport pedagogy. This goes for the learning of movement capability and health but also in relation to outdoor education (OE). The latter a criticized area for having a one-size-fits-all approach to curriculum, with less attention to what to learn in OE, including aspects of everyday practices of being outdoors. The aim of this study was to explore students aged 15 years, and their meaning making of being outdoors expressed in written stories about a favorite place. Two school year eight classes in a Swedish compulsory school situated in an area with high diversity participated. Through this theory-generated empirical study, written stories were explored as one way of evaluating students' meaning making of outdoor places. By using practical epistemology analysis (PEA) to examine experience operationalized through aesthetic judgements attention is paid to the relation between the student and the situation (their favorite place). The analysis make it possible to discern a sense and meaning making of “being” outdoors as an embodied experience, as a relational whole of the self, others and the environment. Descriptions of aesthetic experiences were analyzed leading to dimensions of environing described as “calm and privacy,” “community and togetherness” and “feelings and senses.” A favorite place was by all students described as a very local and nearby place accessible in everyday life. The analysis generated understandings of feelings of “fulfillment” and different embodied experiences of what an encounter with an outdoor place or being outdoors could mean. Furthermore, how personal and diverse the meaning making place tends to be and how experience and habits contribute to the students' creation of microenvironments. Dimensions of environing become part of an embodied process. The analysis of the written stories calls for an alternative understanding of what OE can or should consist of. The findings encourage teachers and researchers to consider alternative understandings and practices of OE that highlight and educate students' overall embodied (individual) experiences and learning in OE and PE.

## Introduction

The development of a re-understanding or reinvestigation of body pedagogy is currently prominent in the research field of physical education (PE) and sport pedagogy (see, for example, Thorburn and Stolz, 2017; Aartun et al., 2020). Several researchers point to regulative discourses restricting or controlling the enabling of transformative approaches to alternative practices and content (Petrie, 2016; Penney et al., 2018). This criticism concerns problematic scenarios lacking awareness of issues connected to inclusion and diversity (Azzarito et al., 2017; Mattingsdal Thorjussen and Sisjord, 2018; Roberts, 2018) but also to traditions of the learning of movement capability and health (Lambert, 2020; Barker et al., 2021; Nyberg et al., 2021) and a growing focus on measurements (Wilkinson et al., 2013; Tidén et al., 2017).

The knowledge area of outdoor education (OE) within PE has been criticized for being a one-size-fits-all approach to curriculum, practice and philosophy and for narrowing learning perspectives (Bond Rogers et al., 2019). Pedagogical units of outdoor techniques have been dominant, together with a long-time ideal of long-established trips and adventure (Backman, 2010; Jeffs and Ord, 2018; Roberts, 2018; Puk, 2021). Less attention has been paid to what to learn in OE, including aspects of everyday practices of being outdoors (Quay, 2013; Mikaels, 2018; Roberts, 2018). OE as a knowledge area within PE also risks positioning students with diverse backgrounds hierarchically and as “the others.” This is due to barriers and existing power mechanisms that benefit white, middle-class values and traditions (Azzarito et al., 2017; Mattingsdal Thorjussen and Sisjord, 2018, 2020). Here studies show that students with diverse backgrounds encounter specific challenges when participating in OE in school due to their families and upbringing lacking outdoor life skills and traditions (Barker et al., 2014; Bond Rogers et al., 2019). At present, there is a need for theory-generated empirical research exploring embodied experiences in OE, which can give reason for a broader conception of OE goals and desired learning outcomes (Bond Rogers et al., 2019). In this paper, we try to unpack what the emotional, physical and cultural aspects of being in a favorite outdoor place look like, what the conditions of the place are and what kind of meanings the students make. Hence, this paper will especially focus on aesthetic experiences as one way of exploring embodied experiences, meaning making and environing in relation to OE and being outdoors.

## A Transactional Perspective with A Focus on Aesthetic Experience

In this paper, we use a transactional framework drawing on the work of Dewey (1934/2005) and Dewey and Bentley (1949/1991), with a focus on aesthetic experience, meaning making and habits, to analyze students' written stories about being outdoors. Here meaning making refers to how experience and habits contribute to the students' creation of microenvironments in which they can pursue and realize their interests through their ends in view (Maivorsdotter and Andersson, 2020). In the active process of creating microenvironments, students incorporate some conditions and disregard others in a process of “environing” (Andersson et al., 2018, p. 100–102). In this way, environing is the process of exploring “encounters” and the experiences of coordinating these into functional wholes (Dewey, 1922/1983, 1934/2005; Garrison, 2001). This transactional understanding of environment is used here as a theoretical lens to explore the emotional, physical and cultural aspects of being in a specific place, in this case being outdoors as part of participating in OE. Being in a particular place creates specific microenvironments in which people develop certain ways of “environing,” expressed in this paper in the students' written stories about their favorite places.

Dewey (1920/1986) uses experience to explain how people are both connected to, and are part of, the world. For Dewey, the fact that people act on the consequences of their own and others' actions in a specific context entails a close connection between what he calls doing and undergoing the consequences of our actions (Maivorsdotter and Wickman, 2011). In these processes, students and outdoor events are continually transformed. As Maivorsdotter and Andersson (2020, p. 1006) put forward: people do not solely witness individual subjects or objects bumping into each other. Instead, they participate in a myriad of transactions between an internal and external environment in which each is connected to the other in particular ways. Just as individual organisms take the external environment into their internal beings through processes of breathing, eating or drinking, students take a particular place into their orientations and habits (see Shilling, 2018). In this article, we try to unpack what the emotional, physical and cultural aspects of being in a favorite outdoor place look like, what the conditions of the place are and what kind of meanings the students make. By taking the dynamic relations between the students and OE as a starting point (Garrison, 2001), meaning is not found in the world itself or captured inside the students' heads. Instead, it is located in the learning activities in which the students are involved. Dewey (1922/1983, p. 16) further explains that “[a]ll virtues and vices are habits which incorporate objective forces.” Hence, being outdoors can be understood as habits incorporating the conditions of the place, bearing in mind that no habit can incorporate its entirety. This means that students need to discriminate and selectively attend to some feelings, interests and problems and disregard others. In a transactional perspective, habits are the tools with which students discriminate and coordinate the continuous flow of experiences into functional wholes (Garrison, 2001). In this paper, we do not observe students' habits but interpret their experiences as dimensions of environing underpinned by habits. We do so by drawing on their written stories. When taking part in OE, the students consequently use resources to make sense of the situation.

### The Significance of Aesthetic Experience

In his work on aesthetics, Dewey (1934/2005) does not seem to significantly differentiate between aesthetic experience and any other kind of experience. Instead, he stresses that every experience has aesthetic qualities perceived as moving toward or away from consummation and fulfillment, that is, whether divergent parts tend to become one whole or not (Maivorsdotter and Lundvall, 2009). But how do we describe an aesthetic experience? According to Dewey (1934/2005), aesthetic experiences are integral, valued and emotional experiences that move toward fulfillment or consummation of ends in view (Maivorsdotter and Quennerstedt, 2012). In this way, we act and feel an anticipated desire for the outcome. Such experiences are not necessarily pleasurable or good but can be something “that is harmful to the world and its consummation undesirable. But it has aesthetic quality” (Dewey, 1934/2005, p. 40). For example, when the students write about their favorite place, aesthetic judgements regarding their emotions and feelings are not only used to share what the place means at the time but also its significance to them. We therefore conduct our research of aesthetic experience through the aesthetic judgements the students included in their written stories. It is in this connection to language that Wittgenstein serves as an addition to Dewey's principle of continuity. Wittgenstein (1967) stresses that the meaning of a word or an utterance is related to its use in a language game that is the activity into which language is woven. Therefore, if we want to explore the meaning of aesthetic experience, we can look at how aesthetic judgements are actually used (Wittgenstein, 1966). Wittgenstein (1966, p. 26) asked us not to concentrate on what aesthetic words like “beautiful” and “fine” represent in order to understand their meaning but rather on how we use them as judgements of taste and what we want. Therefore, in line with earlier studies (Maivorsdotter and Wickman, 2011; Maivorsdotter and Quennerstedt, 2012; Maivorsdotter et al., 2014), aesthetic judgement is operationally identified “as utterances or expressions that either deal with feelings or emotions related to experiences of pleasure or displeasure, or that deal with qualities of things, events or actions that cannot be defined as qualities of the object themselves, but rather are evaluations of taste, for example about what is beautiful or ugly” (Wickman, 2006, p. 9).

The theoretical framework outlined above is used as a theoretical lens to analyze students' written stories, and the analysis draws on this framework. The analysis method—practical epistemology analysis—will be described in depth in the next section.

## The Design of the Study

The presented study is part of a larger project that comes from a government initiative. The purpose of this initiative was to develop models of practice-based research where teachers and researchers meet on an equal footing to decide what problems or challenges could be central to engage in, challenges that, by extension, could contribute to a stronger scientific foundation and a bridging between school, higher education and research. This meant that the outline for this study was a joint action research project. Action research is often described as a research strategy or method used for improving and/or changing practice through collaborative, participatory and cyclic ways in order to produce action, data and knowledge (Carr, 2006; Kemmis et al., 2014). The overall project was accordingly run by a team consisting of not only researchers but also a PETE (Physical Education Teacher Education) educator and a PE teacher. The team started with joint readings and discussions about didactical challenges within OE and focus group interviews with students before designing the actual action research project, whose overall aim, a product of this process, was to explore how to re-image OE within PE by exploring the enabling of alternative practices in peri-urban OE using transformative and place-based pedagogy (in manuscript). A further aim was to explore if and how transformative pedagogy and place-based pedagogy created spaces for reflections on aspects of place, health and well-being.

The planned actions and pedagogical sequences in the overall project were all undertaken in the students' local area. Data was collected continuously of the planned and performed pedagogical sequences, student assignments and evaluations. The iterative cycle contained an evaluation of each sequence and research team meetings leading to both reflections and initiatives about the next phase or step of the planned cycle of action. The pedagogical sequence and student assignment in focus for this specific paper was part of the second phase of the action research project, and is directed toward this empirical material: the written stories about a favorite place and the exploring and analyzing thereof.

### Aim and Sample

The more precise aim of the presented study was to explore 15-year-old students' meaning making of being outdoors as expressed in their written stories about a favorite place. Two Year Eight classes in a Swedish municipally run compulsory school in the region of Stockholm, participated in the study. The municipality has a high diversity in terms of inhabitants with a foreign background; 43%of the inhabitants are foreign born or where both parents are born abroad of Sweden. The housing in the school's catchment area is characterized by rental apartments, privately owned apartments and villas. The school is located <10 min from the central part of the municipality with reachable green areas (parklike area, forest clearings, hills, meadows) and sport fields.

The purposeful sample of the school was determined by the contract that the specific municipality and the university had concluded based on the government initiative and previous collaboration. School year eight was decided by the research team as a relevant age group for this study as this age group is one year from leaving compulsory school, and the Swedish PE-syllabus opens up for a focus on peri-urban outdoor teaching and learning for this age group. One of three central areas is *Outdoor life and Activities* where the aim is to stimulate students to acquire knowledge of being outdoors as part of cultural traditions, but also in terms of relevant clothing, safety aspects, rules of right of public access (SNAE, 2011). The other two central areas are *Movement and Health and lifestyle*. The PE teacher who had registered an interest in participating in a project about peri-urban OE and became part of the project team. The teacher was the one that decided what classes would be suitable for the project according to the school and the teacher's overall planning of PE.

### An Action Research Approach

An important part of an action research study is deciding upon the theoretical framework for the planned actions. Here a transformative perspective, together with a place-based pedagogy, was suggested by the responsible researcher to guide the pedagogical actions and enable a focus on transformation and transactions in relation to local place and aspects of health and well-being. Adopting a transformative perspective was justified as one way of challenging existing PE and OE discourses. This perspective links to social constructivism, pragmatism and experiential learning (Dewey, 1934/2005). A transformative learning perspective is defined by Mezirow (2018) as “a process by which we transform problematic frames of reference (e.g., mindsets, habits of mind, meaning perspectives)—sets of assumptions and expectations—to make them more inclusive, discriminating, open, reflective and emotionally able to change” (p. 116). This critical perspective enables students to recognize and reassess structures of assumptions and experiences that frame their thinking, feeling and acting (Meerts-Brandsma et al., 2020). It involves cognitive, social, moral and affective components as well as values of structural and cultural conflicts because structures of meaning construct references (Mezirow, 2018). When converted into a pedagogy, namely transformative pedagogy, this perspective relates to a dialogic construction of meaning, tolerance and authentic educational experiences, where the learning process aims at transformation and not only adaption (Ukpokodu, 2009, 2011). Adopting a transformative perspective means there are no rights or wrongs; instead, to complete the assignment, the students are encouraged to independently question, discuss, reason, think and act.

An important aspect of adopting a transformative perspective and pedagogy is to go from teacher- to student-centered teaching and as a teacher to work more as a facilitator and a guide. This meant that the students became co-constructors of their knowledge acquisition. The “action” in terms of perspective and the choosing of student assignments helped the teachers to increase the students' ability to engage in learning processes and to take charge of their assignment and learning processes (Dewey, 1934/2005; Mezirow, 2018).

The tenets of place-based pedagogy were used for the design of teaching actions often employed in experiential learning, with a focus on the local environment through a series of visits to nearby environments (Boyes, 2000; Gruenewald, 2003; Wattchow and Brown, 2011; Brown, 2012). Place-based OE is advocated as a way to enable an awareness of how we relate to places, the cultural history of a place and how places relate to us (see, for example, Hill and Brown, 2014; Mikaels, 2018).

### Data Production

As part of the assignments given, the students were asked to write a short story (not more than one page) about their favorite outdoor place (what characterizes the place, what makes it special to you, and what happens when you visit this place). The students were free to choose the place and were not given any other instructions. The aim of the assignment was to work with the students' awareness of place linked to aspects of being outdoors and everyday life. The students sent in their stories to their PE teacher through the school's learning platform. During the collection period for the stories, there was a high rate of absence in the classes due to Covid-19 pandemic. In spite of the pandemic, all compulsory schools in Sweden were held open. All PE-lessons were organized outdoors and no changing of clothes were required. But as the pandemic was present, this led to a higher amount of students being absent from school and PE classes. In total, 24 stories (14 by girls and 10 by boys) were collected and analyzed out of 28 available for this specific study. The excluded stories (two by girls and two by boys) did not include experiences of being outdoors. The total number of students in the two classes was 47.

### Data Analysis

The analytical approach was based on how people make meaning with text. It drew on the work of Rosenblatt (2005), combined with practical epistemology analysis (PEA) developed by Wickman and Östman (2002) and used by Maivorsdotter and colleagues in several studies (Maivorsdotter and Wickman, 2011; Maivorsdotter and Quennerstedt, 2012; Maivorsdotter et al., 2014).

#### Ethical Considerations

The ethical considerations undertaken included both to present the aim of the overall project and get the permission to conduct the study from the principal of the school, and to obtain a written consent form by the students and students' caretakers. In the information letter to the students and caretakers, the purpose and methodology of the study was presented, as well as information of the securing of anonymity (pseudonymization), how the data and findings would be used and that the students could withdraw from the study at any time. Contact information to the teacher and responsible researcher was also available in the letter. No student or student's caretaker asked to withdraw from the study.

#### Making Meaning With Text

In this paper, meaning making was seen as a process where the elements of analysis are actions situated in communication and in whole activities. From this perspective, writing was seen as action because when we write something, we do something that has both a purpose and a consequence as part of an activity (Maivorsdotter and Quennerstedt, 2012). However, to understand the writing, we also had to grasp the situation in which transactions occur. Any description of an experienced situation first needs to include which kinds of elements were encountered in the experience before we could describe the transactions that occur (Maivorsdotter and Wickman, 2011). In this case study, the elements were underpinned by the larger practice-based project designed to re-image OE through transformative and place-based pedagogy. Taking part in this overall project influenced the students' writing about their favorite place as well as the researchers' understanding of the students' stories.

#### Practical Epistemology Analysis

PEA is designed to analyze the direction that learning and meaning making take as a result of situated transactions occurring in educational situations (Wickman and Östman, 2002; Wickman, 2006; Andersson et al., 2018). PEA is undertaken in several steps, and the researcher moves back and forth between the steps during the analytical process.

In this study, the purpose was to explore aesthetic experience in OE within PE and how students were environing the world aesthetically as part of being outdoors. What we were looking for in the students' stories were descriptions of outdoor events demarcated by the aesthetic qualities expressed in terms of moving toward or away from the fulfillment of the aesthetic quality of the experience or, in other words, events where aesthetic expressions had a place. Dewey (1934/2005) argued that

…in discourse about an experience, we must make use of these adjectives of interpretation. In going over an experience in mind after its occurrence, we may find that one property rather than another was sufficiently dominant so that it characterizes the experience as a whole. (p. 38)

In his arguments about aesthetics, Shusterman (2007) described them as evaluative colored adjectives. We therefore searched the students' stories for events of aesthetic experiences that through evaluatively colored adjectives (e.g., aesthetic judgements) moved toward or away from the fulfillment of being outdoors. The first step in the analysis was consequently to distinguish outdoor events in the students' stories as operationally defined in the paper.

In the second step, PEA was used as follows (i–iv): firstly, to identify the (i) *ends in view* in the selected outdoor events. These ends in view were influenced by the purpose of the study and could be summarized as “to be in a place fulfilling aesthetic qualities of being outdoors.” This major theme was then categorized into minor themes, namely “a place near home,” “a place near friends or loved ones” and “a scenic place.”

Secondly, having identified the minor ends in view, the next step in the analysis was to identify (ii) *gaps* that emerged from these ends in view. For example, if the end in view was to experience aesthetic qualities in being outdoors near to home, this opened up a gap between the fulfillment and non-fulfillment of aesthetical qualities according to the student's taste for outdoor events. To continue the story, the student therefore had to fill the gap of fulfillment or non-fulfillment of aesthetical qualities with relations.

Accordingly, the next step in the analysis was to identify the various kinds of (iii) *relations* that the students used to fill the identified gaps. Studies (Jacobson and Wickman, 2008; Maivorsdotter et al., 2015) show that students use aesthetic judgements when making meaning of an experience as a move toward fulfillment (having a positive aesthetic experience) or a move away from fulfillment (having a negative aesthetic experience). For example, if the student used the aesthetic judgement “feeling great,” we can see that they experienced sitting on a bench with friends in the back garden as moving toward the fulfillment of being outdoors. On the other hand, if they used the aesthetic judgement “it was too boring,” we can see that they experienced sitting on the bench as moving away from the fulfillment of being outdoors. Aesthetic judgements thus provide us with information about how the students viewed their experience of taking part in a specific event. In this analytical context, a *relation* is a statement or utterance that construes a connection between the entities of experience. A relation concerns what the case is or how certain things are valued. Values are often aesthetic judgements about desires or taste. For example, if the student used positive aesthetic judgements about sharing a meal with friends in the park, this relation shows what kind of behavior they valued in this situation. Finally, the identified relations were sorted into dimensions of environing, in this study called “calm and privacy,” “community and togetherness” and “feelings and senses.”

In constructing relations, the students also described some of the things encountered in these experiences. Identifying (iv) *encounters* was therefore the last step in the analysis. For example, the encounters could involve friends, fresh air, family habits or the students' previous experiences. The relations construed when the students described different encounters illuminate the connection between the student and the situation as a whole.

## Findings

Our analysis identified the theme “to be in a place fulfilling aesthetic qualities of being outdoors” as the major end in view in the students' stories. This major theme was then categorized into minor themes, namely “a place near home,” “a place near friends or loved ones” and “a scenic place.” These themes, aesthetic experiences and dimensions of environing will be elaborated below. The names of the students are fictitious, and the italicized quotations are our own. All the quotations have been selected based on their representativeness of the theme.

### A Place Near Home

The theme, A place near home, includes ends in view about being in a place near home that fulfills the aesthetic qualities of outdoor events, according to the storytellers. In the stories, “a place near home” can be the balcony, the back garden or a small forest just outside the block of flats, but it can also be a place in the student's homeland. In this theme, the favorite place is characterized as familiar and “near,” either near in terms of being close to where they live or near in terms of being the roots in its place of origin. The theme will be illustrated in two quotations where Mira and Rúben describe their favorite outdoor places—the balcony and a small city forest.

**Mira**

(1) Sometimes when *I'm bored* (especially during the night) or when *I'm feeling unwell*, I walk

(2) out onto our balcony to get some fresh air. The fresh air *feels fresh*, and I'm in a *good mood*

(3) in a couple of minutes. Going out onto the balcony is also what I usually do during the

(4) summer when I wake up at four or five o'clock. The birds, the sun and *the soft breeze*, and

(5) *the summer feeling is great* and then *I feel much better* and hopefully can fall asleep again. I

(6) usually spend most of my time on the balcony during the summer when it is sunny, warm

(7) and most *comfortable (nice)* to be outdoors. My family and I usually eat breakfast and listen

(8) to calm music to start the day *feeling good* and motivated. When we invite family over, it is

(9) like so *cozy to sit and cuddle* on the balcony. Sometimes when my family is sleeping, and I

(10) don't want to wake them when I'm talking on the phone, I usually go out on the balcony

(11) and talk so that the sound doesn't carry to them. Therefore, I feel my balcony *is the perfect*

(12) *place to be* :).

This story includes an *end in view* about being in a place near home that fulfills aesthetic qualities of outdoor events, according to Mira. This purpose opens up *a gap* in the story concerning how aesthetic qualities of outdoor events are fulfilled in this place. Mira fills this gap with *relations* expressing positive aesthetic experiences. In the beginning of the story, Mira uses the aesthetic judgements “being bored” or “feeling unwell” (1) and how these feelings are transformed into “a good mood” by the fresh air on the balcony (2). She continues her story by using the aesthetic judgements' “the soft breeze” (4) and “the summer feeling” (4). These aesthetic experiences show that being alone on the balcony and feeling the breeze and the sunlight are experiences of the fulfillment of being outdoors. In the next lines, Mira tells us that these expressions also are present when she is with her family. She does so using the aesthetic judgement “feeling good” (7–9) to describe having a family breakfast on the balcony or relaxing with relatives. Mira ends her story with a final aesthetic judgement summarizing her experience of outdoor events on the balcony— “it is the perfect place to be” (11, 12). Mira's story contains several physical encounters like the balcony or the telephone but also encounters with nature in terms of birds, sunny weather and fresh air. The story includes as well-cultural encounters such as a family breakfast, meeting relatives or the need for privacy (night-time phone calls on the balcony).

In this short story, Mira describes all hours of the day, from the night-time through to the morning and daytime and then back to the night-time again. Her story also shows how by being on the balcony, she is environing in multifaceted ways. Mira's story highlights an intrinsic combination of *calm and privacy* and *community and togetherness* as aesthetic qualities in outdoor events. She uses the balcony to be alone and to have a gentle start to the day with her family as well as for events of a gentle communion with her relatives. At night, when her family are asleep, Mira can experience privacy on the balcony. But at the same time, she takes part in a community outside the family when talking to someone on the phone. In this story, *feelings and senses* are also clearly a dimension in the identified process of environing. Mira tells us about feeling in a good mood and being motivated as well as the sensations of fresh air, a soft breeze, sunny weather and soft music.

In the following story, Rúben's description of his favorite place is not his flat but a section of forest just outside his flat. Rúben expresses the fulfillment of being outdoors as having a place that feels like “his own.”

**Rúben**

(13) My *favorite place* is out in the forest, and this place is right outside my flat. (14) What makes it *my favorite place* is that there is a bench where I usually sit when

(15) I come to this place. Around me are lots of trees and bushes but also

(16) animals such as birds flying around. *At my favorite place*, there is also a path that leads (17) to a small lake that I usually walk past when I do certain errands. But if

(18) you continue back down that path, you end up at my flat. But what makes the

(19) place so special to me is that *it feels like my own special place*. Usually not

(20) many people pass by, and that makes it usually *calm and quiet there*. I myself have never

(21) had my own place, so when I come to my favorite place, *it really feels like this place is my*

(22) *own*. And every time I come to my special place, *I always feel so peaceful*. So if I, for

(23) example, have had *a tough day* and come to that place, *I usually feel very calm*. It also

(24) allows me to really concentrate on my homework that I will do later in the day.

The story begins with a description of what Rúben's favorite place looks like and where it is located in relation to his home. Rúben also tells us what he usually does at this place. He adds an aesthetic quality to the description by calling the place “my favorite” (13, 14). The quality of the place and his actions (sitting on a bench, watching nature and animals, walking beside a small lake) are outdoor events that lead to the fulfillment of his taste for being outdoors. In the second part of the story, Rúben uses the aesthetic judgements “I always feel so peaceful” (22) and “I usually feel very calm” (23). Being alone, having privacy and experiencing calm and quietness are aesthetic qualities that Rúben expresses as leading to the fulfillment of being outdoors. This experience has clearly aesthetic qualities that Rúben condenses into a single statement: it feels like this is his own special place (19, 21, 22).

Rúben's story includes the *end in view* about being in a place near his home that fulfills the aesthetic qualities of outdoor events. This purpose opens up *a gap* in his story concerning how aesthetic qualities of outdoor events are fulfilled in this place. Throughout his story, Rúben fills this gap with *relations*, which we have outlined in the section above. By sitting on the bench in the small forest outside his home, Rúben creates a microenvironing that supports dimensions of environing in terms of *calm and privacy* and *feelings and senses*. The encounters expressed in his story can be summarized as urban nature and the absence of other people. These encounters are central conditions in Rúben's environing of being outdoors.

### A Place Near Friends and Loved Ones

This theme includes ends in view about being outdoors, with a focus on social relationships that fulfill aesthetic qualities of outdoor events, according to the students. In their written stories, “a place near friends and loved ones” can be the local football club or the community playground. In this theme, the favorite place is characterized as one that supports social actions and good relations with others. The actions can be organized as in the case of the football club or spontaneous like at the community playground. The theme will be illustrated in two quotations where Karim and Zelina describe their favorite outdoor places with a focus on friends and loved ones.

Karim's favorite place is the local football club. He describes this regular outdoor event by using a number of aesthetic judgements. In Karim's storytelling of being outdoors, the *end in view* is being a successful part of a social event or, in other words, being a player in a football team. This end in view opens up a *gap* that Karim fills with *relations*, including the community and togetherness dimension of being outdoors.

**Karim**

(25) My *favorite place* is the football field called X-dike. It has about seven

(26) changing rooms, a café and two football pitches. X-dike is my *favorite place*

(27) because I *love* football and am there about 5–6 times a week. I *feel at home*

(28) there, and I *feel safe*. I also have many friends who are usually there often.

(29) My *favorite place* represents football. Almost every day there is a lot going on on the

(30) pitches with training sessions and matches. Every weekend, different teams usually come

(31) and play against our teams. I often play matches, but if I don't, I usually watch.

(32) The X-dike *feels like mine* because football is always played there, which I *love*.

(33) When I'm in my favorite place I *feel happy* and I *am energized to play well*.

(34) That's why X-dike is my *favorite place*.

Karim's story begins with some aesthetic judgements: his favorite place (25) is the local football pitch because he “loves” (27) football and therefore visits several times a week. This is a social event that he shares with his friends at the club; they also spend a lot of time on the football pitch (28). Karim confirms that playing football with friends is an important aesthetic experience. Taking part in this social outdoor event makes him “feel at home” (27) and “feel safe” (28). Karim puts forward that the characteristics of football—football pitches being used (30), training and matches (30), local football teams and other teams (30, 31), weekdays and weekends (29, 30), playing or watching football (31)—have positive aesthetic connotations. Like Rúben, in the analysis above, Karim experiences X-dike as his place; it “feels like mine” (32). He loves football, and this is a place dedicated to this outdoor event. X-dike is a microenvironment where Karim “feels happy” (33), and the conditions at X-dike “energize” him (33) to fulfill his interest in being outdoors namely to be a better football player. Throughout his story, Karim fills *the gap* of being a successful part of a football team with *relations*, including the football community and positive social relations. Important *encounters* in this microenvironment are friends sharing Karim's interest in football, the football club facilities and the football culture.

In the following story, Zelina writes about her favorite place, mainly focusing on social meetings even if she also tells us about being alone. Her story includes an *end in view* about being in a place that offers and supports social relations outdoors. For example, when Zelina is alone in the playground, she notices other children and feels happy when they laugh. The end in view in her story opens up *a gap* concerning how aesthetic qualities of outdoor events are fulfilled in this place. Zelina fills this gap with *relations* expressing several positive aesthetic experiences. For example, relaxing with a friend without a screen (calm and privacy), or sharing snacks and talking about life (community and togetherness), or enjoying the urban nature and seasonal changes (feelings and senses).

**Zelina**

(35) […] The playground is surrounded by flats and a road that is quite close to a school

(36), so when I sit there, many children usually walk by and laugh with their friends, who also (37) usually make me a little *happy*. The park also has very nice trees that are very green in the

(38) summer but also *nice* in the autumn when they turn orange, purple and yellow. It is

(39) especially *very nice* during warmer seasons, and because there are many trees around us,

(40) the air is very *clean and refreshing*. Sometimes I just like to take a big breath.

(41) I also *feel very safe* because it is a fairly quiet and bright area.

(42) The sun isn't covered by something, so it is almost always bright and clear.

(43) Because it is close to Coop [a supermarket chain], I usually go and buy something like

(44) crisps or a drink and sit there and eat it usually with a friend.

(45) Then we usually sit there and just talk about things that happened in school, on the

(46) internet or just generally in our lives. *This place is also good* because where we usually sit,

(47) there is a roof that both protects us from the sun and provides shade on hot days but

(48) also protection from the rain on rainy days. So we can go there any day after school.

(49) I tend to be *very relaxed and happy when I sit there*, mostly because I sit with my friend

(50) and talk and laugh but also because I am sitting in nature instead of being at school or

(51) on a screen, e.g. FaceTiming. I don't know why, but nature makes me feel *more “alive*”

(52) if you can put it like that. It makes me *happier*. Therefore, I like to go there after a long

(53) day at school, sometimes with my friend, *to relax or just to have fun*.

Zelina begins her story by describing her favorite place—a playground in the local community. She then uses the aesthetic judgement “happy” (37), showing that children laughing is an experience leading to the fulfillment of being in the playground, even if she is sitting alone. In the next lines, Zelina uses a number of aesthetic judgements (37, 38, 39, 40) to show how the seasonal experiences of trees and fresh air fulfill the experience of being outdoors as well. The playground is rather quiet and open, which makes her “feel safe” (41). The place also offers shelter from sunlight and rain, something that Zelina feels is “good” (46–48). She and her friends can visit the playground every day without needing to worry about the weather. At the end of her short story, Zelina highlights some aesthetic experiences that seem to be of particular importance since they characterize this microenvironment. Being outdoors at the playground makes her feel “relaxed” and “happy” (49). Zelina describes how she shares this microenvironment with a friend and how her mood in some way changes when being outdoors, away from school and digital screens, and she becomes happier. Zelina tries to grasp this experience of outdoor events by using the aesthetic judgement “more alive” (51). Being outdoors at a place supporting social relationships, including face-to-face meetings, seasonal changes and fresh air, makes her happy (52) and relaxed (53).

### A Scenic Place

The third identified theme consists of ends in view related to the scenic aspects of the students' favorite places, for instance a public park, not necessarily in the local area, still within the larger municipality, or a cottage in another part of the country but “local” in terms of being family property. In the stories, the scenic aspects of being outdoors are greenery and wildlife, sounds of water and feeling the wind and the fresh air. The greenery can be cultivated (like in the public park) or wild (for example all around the cottage) or something in between. Animals add aesthetical qualities to the scenic places in the stories, and they range from ants to birds and cats. “Wild” animals like bears and foxes are not included in these microenvironments. The two stories outlined in this section are written by Sonya and Malik. The analysis shows how different aspects (emotional, physical and cultural) of being at a scenic place are interwoven in complex ways.

In the following, the *end in view* is the fulfillment of being outdoors at a scenic place. The microenvironment in this story is not any particular place. Instead, Sonya tries to grasp general aspects of greenery by using the concept “nature reserve.” This end in view opens up a *gap* in Sonya's story that she fills with *relations* expressing two dimensions of outdoor events, namely “community and togetherness” and “feelings and senses.” Sonya is one of the few students in our study who include a negative aesthetic experience in their story. According to her, the nature reserve being blighted by litter is a negative aesthetic experience leading away from the fulfillment of being in a scenic place.

**Sonya**

(54) My favorite place is not a specific place, just a kind of area that I like to be in; it

(55) is a nature reserve, not a specific nature reserve, just anyone I have been to.

(56) The reason that it is a nature reserve (I am writing about) is also that I think these

(57) are *very beautiful places*, and it is just special when you are out with family or

(58) friends in nature because it feels like *all relationships with those people greatly improve*

(59) when you are out in nature, and you can make new relationships in

(60) nature. When I'm at a nature reserve it makes me *very happy*, and I would never want to

(61) lose it; so when I see any rubbish on the ground in nature I always pick it up and

(62) I try to *be as good to nature as I possibly can*.

The story begins with the aesthetic judgement “my favorite place,” followed by a clarification that the “place” is not necessarily a specific physical place but a place with specific aesthetic qualities. It has to be “a very beautiful place” (57). In her story, Sonya seems to argue that beautiful places should be protected; in line with this assessment, Sonya uses the words “nature reserve” (55) to describe her favorite place. Sonya's short story contains an intrinsic combination of the dimensions “community and togetherness” and “feelings and senses.” In the middle of her story, Sonya tells us how she experiences relations with family and friends as developing and deepening when she socializes with them outdoors (58, 59). The *encounter* with nature seems to influence her social life in a way that Sonya appreciates. This aesthetic experience influences her actions. She is also careful not to lose this way of socializing (with nature) and therefore picks up rubbish whenever she sees it (61). Sonya ends her storytelling with the aesthetic judgement “I try to be as good to nature as I possibly can” (62), a judgement showing how she values the scenic aspects of being outdoors.

In the following story, Malik takes us with him to his family's cottage in another part of the country. The *end in view* in the story is being in a place fulfilling the aesthetic qualities of a scenic place. This purpose opens up a *gap* that Malik fills with *relations* expressing positive and negative aesthetic experiences.

**Malik**

(63) My favorite place is in Y-place, by my family's summer cottage. It is *incredibly nice* there,

(64) and we usually go there in the summer. There is a large open terrace with a hammock and

(65) a view of a lake. There are also plantations there and a forest. When I'm there

(66) *I feel incredibly good*. I am not someone who goes on holiday a lot, but when I do

(67) I *rarely feel really calm*. In Y-place, it is different. We usually sleep in a hotel (me and my

(68) family), but we go out after breakfast. When I come to the cottage I become so *happy and*

(69) *calm*. But who wouldn't be? I mean, the birds are chirping, the family cats are walking a

(70) little bit here and there and the air is cool.

Malik uses the aesthetic judgements “favorite place” (63) and “incredibly nice” (63) to describe the fulfillment of being outdoors at this place. The scenic aspects highlighted by Malic are the large terrace, the lake view, the surrounding plantations and the forest nearby: all positive conditions in this microenvironment. Malik summarizes his experience by using the aesthetic judgement “when I am there I feel incredibly good” (65, 66). This is a positive aesthetic experience leading to the fulfillment of being outdoors; however, in the next line, Malik tells us about the opposite feeling. Traveling to the cottage in northern Sweden is a positive aesthetic experience. However, Malik is not a seasoned traveler, and he rarely feels relaxed (66, 67). Taking part in this kind of activity is normally a negative aesthetic experience for him. In this way, Malik sheds light on an important aspect of participating in outdoor events—the activity has to be familiar in some way. All the aspects of being outdoors identified in this study (calm and privacy, community and togetherness and feelings and senses) are perfectly interwoven at his family's cottage. Malik ends the story by writing “when I arrive at the cottage I become happy and relaxed” (68, 69). “But who wouldn't be?” (69) he asks himself. According to Malik, encountering birds, cats and fresh air are aesthetic experiences clearly fulfilling the end in view of being outdoors.

## Discussion

The more precise aim of this study was to explore the meaning making of 15-year-old students at a Swedish upper secondary school with regard to being outdoors as expressed in their written stories about a favorite place. The written stories were the outcome of a student assignment during a lesson on peri-urban OE belonging to a larger action research project in which the students with their teacher participated. The project was designed to re-image and enable alternative practices in OE within PE. This also involved exploring if and how transformative pedagogy and place-based pedagogy created spaces for reflections on aspects of place, health and well-being. In the presented study we have unpacked through the PEA method emotional, physical and cultural aspects of a favorite outdoor place highlighting what the place was like and what kind of meaning making the students made.

The PEA analysis of the written stories identified the theme “to be in a place fulfilling aesthetic qualities of being outdoors” as the major end in view in the students' stories. Categorizing this major theme into minor themes generated three themes: “a place near home,” “a place near friends or loved ones” and “a scenic place.” Descriptions of aesthetic experiences and dimensions of environing were analyzed for each minor theme. The identified dimensions were “calm and privacy,” “community and togetherness” and “feelings and senses,” thus giving us as readers a sense of embodied taste for the chosen and described favorite place. The findings help us to notice how the described aesthetic judgements, feelings or emotions are related to experiences of pleasure or displeasure and how this can be seen as evaluations of taste (Wickman, 2006, p. 9). This, in turn, gives us as researchers (and teachers) knowledge of what kinds of gaps and relations occur that may hinder or support different learning processes, leading to possible encounters and meaning making. The analysis generated understandings of feelings of “fulfillment” and different embodied experiences of what an encounter with an outdoor place or being outdoors could mean. Furthermore, the findings highlight how personal and diverse the meaning making of the embodied experiences of a favorite outdoor place tends to be. Here meaning making refers to how experience and habits contribute to the students' creation of microenvironments (their favorite place) in which they can pursue and realize their interests through their ends in view (Maivorsdotter and Andersson, 2020). In the active process of creating microenvironments, students incorporate some conditions and disregard others in a process of “environing” (Andersson et al., 2018). This process has aesthetical connotations and expresses a specific “taste” for being outdoors. Drawing on this method, we found that teachers and students, as part of teaching and learning processes in OE, need to pay attention to the value of both considering and taking into account different tastes of being outdoors (Mezirow, 2018). This is to critically engage in and open up for reflections challenging frames of reference (mindsets), habits of mind and meaning perspectives in OE within PE (Backman, 2010; Jeffs and Ord, 2018; Roberts, 2018; Mattingsdal Thorjussen and Sisjord, 2020; Ho and Chang, 2021).

The analysis of the written stories calls for an alternative understanding of what OE can or should consist of. The majority of the students chose to describe and identify places close by, valuing accessibility and simplicity in relation to a favorite outdoor place and being outdoors. The students use what is familiar and of value in their creation of a favorite place. There are no stories about places representing remote nature places, like visits to the mountains, the sea, or wilderness landscapes. A favorite place is described as a very local and nearby place accessible in everyday life. Furthermore, the stories also describe positive aesthetic judgements of being outdoors.

What was clear from the planning of the overall project was that the students' experiences of outdoor life did differ, from no experience to often practicing outdoor life. From this small scaled study we are careful not to draw any conclusions of how gender, socio economical or cultural background affected the students' writings. Some of the written stories in the study, from newly arrived students, describe favorite places in their home countries. Getting an assignment to write about a favorite outdoor place seems to activate memories of a geographic place that feels familiar. This is understandable in many ways and can be understood as part of a migration process where multiple identities are available (Azzarito et al., 2017). What needs to be noted is that the identified themes did not, however, differ from the other students' stories.

The overall findings throw up questions of didactical implications for PE teachers: what can a favorite outdoor place be and how can relations to places be highlighted as part of learning and making meaning of being outdoors. The written stories emphasize an openness for simplicity and often a non-sportive outdoor engagement as part of lifelong learning in relation to place, nature and well-being. The students find or create their own microenvironments and use aesthetic judgements when making meaning of an experience as a move toward fulfillment (having a positive aesthetic experience) or a move away from fulfillment (having a negative aesthetic experience). In the literature, dominant goals and values of being outdoors and OE within PE are often combined with adventurous activities, safety and equipment (see, for example, Mikaels, 2018; Roberts, 2018; Bond Rogers et al., 2019). These prevailing discourses risk excluding students with less OE experiences, skills and/or traditions (Barker et al., 2014; Azzarito et al., 2017; Mattingsdal Thorjussen and Sisjord, 2018, 2020). Observing aesthetic judgements has thus provided us as researchers and teachers with information about how the students viewed their experience of being outdoors in their favorite place. It also provides us with possibilities to open up for our own reflexivity and for educative inquiries of meaning making related to being outdoors and as part of a learning process in relation to experiences of OE (Dewey and Bentley, 1949/1991, p. 521). We as researchers were affected by the students' openness to share their favorite places. What struck us was how our own expectations of what could count as a place outdoors in the context of teaching and learning of the outdoors differed. One of us has a background as a PE-educator and one of us has a preschool teacher exam. Being educated in PE with a more inflexible frame of what being outdoors can mean was challenged in terms of what kinds of dimensions of environing that the students actually described and valued.

These findings give reason for a rethink and a broader conception of OE goals and desired learning outcomes. The action-based study and the use of transformative pedagogy encouraged the students to think and act independently. This, together with the place-based pedagogy, meant that the students also read and related to the place. The value of incorporating spaces and assignments for reflections when teaching OE is also part of the study's findings. In the overall evaluation of the larger study, the students commented that they enjoyed being in charge of, for example, this writing assignment about a favorite place; they “owned” the assignment and felt that this was empowering (in manuscript).

### Conclusions and Limitations of the Study

The analysis of the written stories about a favorite outdoor place make it possible to discern a sense and meaning making of “being” outdoors as an embodied experience, as a relational whole of the self, others and the environment (Quay, 2013). One conclusion from the findings is that exploring and sharing a favorite outdoor place can become part of both an experience and a reflexive learning process where place touches upon aspects and relations of well-being but also on cultural, ecological and/or historical readings. Habits seen as transactions of experiences and dimensions of environing become more than just learning about a physical place or a technical OE activity; they become part of an embodied process. This type of student assignment may support teaching and learning processes that highlight and educate students' overall embodied (individual) experiences and learning in OE and PE (Thorburn and Stolz, 2017; Puk, 2021).

The findings from the study challenge teachers' views on what an (ideal) OE should consist of and what the individual experiences can or should be (Backman, 2010; Jeffs and Ord, 2018; Roberts, 2018; Puk, 2021). In a longer perspective, an interest in students' habits, past experiences and tastes can support and contribute to other more inclusive forms of practices.

Through a theory-generated empirical study, we have closely explored written stories as one way of evaluating students' meaning making of outdoor places. This was done by drawing on their experiences and habits of life. We are well-aware that this is a small study with an analytical method very rarely used by researchers interested in OE within PE. A qualitative study's trustworthiness is dependent on several aspects such as rigor, sincerity, credibility as well as resonance, coherence and transparency (Tracy, 2010). In this small scaled study we have worked closely in relation to the empirical material, described each step taken to make the analysis transparent, making it possible for the reader to follow the process and the coherence of the findings, what claims made and how we claim them. Furthermore the analysis was conducted by a researcher who had designed the larger project, together with a researcher who had not been involved in the project. This was to get an “inside” and “outside” perspective on the students' stories, and to strengthen the rigorousness and credibility of the analysis. A limitation of the study is the small sample and the high absence rate of the students due to the pandemic. Here we cannot say in what ways this effected the study's final findings. Furthermore, we lack detailed knowledge of the students' families' socio-economic status and cultural background, which suggests that further studies are needed to examine how these factors and/or gender may influence students' experiences of and stories about a favorite outdoor place.

Our concluding thoughts are that the findings encourage teachers and researchers to consider alternative practices to include a broader learning perspective and open up for students' everyday experiences of being outdoors. This is to re-investigate and/or re-image the educative role of OE within PE as part of a lifelong learning and embodied experiencing of being outdoors.

## Data Availability Statement

The raw data supporting the conclusions of this article will be made available by the authors, without undue reservation.

## Ethics Statement

Ethical review and approval was not required for the study on human participants in accordance with the local legislation and institutional requirements. Written informed consent to participate in this study was provided by the participants' legal guardian/next of kin.

## Author Contributions

SL and NM have both contributed to the writing of the original draft preparation and finalized findings and conclusions. SL has had a special responsibility for the data collection and introduction and discussion. NM has been responsible for the formal methodology and analytical framework. All authors contributed to the article and approved the submitted version.

## Funding

This research has received funding from The Swedish Research Council for Sport Science (P2019-0164). There is no conflict of interest in terms of the study design, data collection, analysis, and interpretation of the results, Funding for the open access publication fee is received from the funding of the project (The Swedish Research Council for Sport Science) and the Swedish School of Sport and Health Sciences.

## Conflict of Interest

The authors declare that the research was conducted in the absence of any commercial or financial relationships that could be construed as a potential conflict of interest.

## Publisher's Note

All claims expressed in this article are solely those of the authors and do not necessarily represent those of their affiliated organizations, or those of the publisher, the editors and the reviewers. Any product that may be evaluated in this article, or claim that may be made by its manufacturer, is not guaranteed or endorsed by the publisher.
